# Efficacy and safety of bilateral continuous theta burst stimulation (cTBS) for the treatment of chronic tinnitus: design of a three-armed randomized controlled trial

**DOI:** 10.1186/1745-6215-10-74

**Published:** 2009-08-21

**Authors:** Carola Arfeller, Reinhard Vonthein, Stefan K Plontke, Christian Plewnia

**Affiliations:** 1 University Hospital of Tübingen, Department of Psychiatry and Psychotherapy, Osianderstraße 24, 72076 Tübingen, Germany; 2 University Hospital of Tübingen, Department of Medical Biometry, Westbahnhofstraße 55, 72070 Tübingen, Germany; 3 University Hospital of Tübingen, Department of Otolaryngology, Elfriede-Aulhorn-Straße 5, 72076 Tübingen, Germany

## Abstract

**Background:**

Tinnitus, the perception of sound and noise in absence of an auditory stimulus, has been shown to be associated with maladaptive neuronal reorganization and increased activity of the temporoparietal cortex. Transient modulation of tinnitus by repetitive transcranial magnetic stimulation (rTMS) indicated that these areas are critically involved in the pathophysiology of tinnitus and suggested new treatment strategies. However, the therapeutic efficacy of rTMS in tinnitus is still unclear, individual response is variable, and the optimal stimulation area disputable. Recently, continuous theta burst stimulation (cTBS) has been put forward as an effective rTMS protocol for the reduction of pathologically enhanced cortical excitability.

**Methods:**

48 patients with chronic subjective tinnitus will be included in this randomized, placebo controlled, three-arm trial. The treatment consists of two trains of cTBS applied bilaterally to the secondary auditory cortex, the temporoparietal associaction cortex, or to the lower occiput (sham condition) every working day for four weeks. Primary outcome measure is the change of tinnitus distress as quantified by the Tinnitus Questionnaire (TQ). Secondary outcome measures are tinnitus loudness and annoyance as well as tinnitus change during and after treatment. Audiologic and speech audiometric measurements will be performed to assess potential side effects. The aim of the present trail is to investigate effectiveness and safety of a four weeks cTBS treatment on chronic tinnitus and to compare two areas of stimulation. The results will contribute to clarify the therapeutic capacity of rTMS in tinnitus.

**Trial registration:**

The trial was registered with the clinical trials register of  (NCT00518024).

## Background

### Tinnitus

Tinnitus is the perception of sounds or noise in the absence of an external stimulus. About 10 to 15% of the general population [1] report this auditory phantom perception [2]. Around 1 to 2% of the patients are seriously impaired [1]. Sleep disturbances, depression, irritability, and anxiety symptoms are the most common psychiatric comorbidities of tinnitus [1,3]. Current therapies focus on the "management of tinnitus", i. e. they are a means to reduce tinnitus perception or awareness, and treating comorbidities rather than curing tinnitus itself [4-6]. Cognitive-behavioural therapies yield some relief [7], but are often not sufficient. To date, there is no pharmacological intervention or device available that has been proven to reliably reduce tinnitus. The pathophysiological causes of tinnitus are poorly understood which impedes the development of rational and evidence based therapies. However, recent findings indicate that chronic tinnitus is the result of maladaptive reorganization in the central auditory system [8] that can be reflected in hyperacitivity of cortical areas involved in the perception and processing of auditory information [6,9-14].

### Transcranial magnetic stimulation

Transcranial magnetic stimulation (TMS) is a non invasive method to depolarize cortical neurons based on the principle of electromagnetic induction [15]. Applying series of rapid consecutive single stimuli is called repetitive TMS (rTMS). Low frequency rTMS (1 Hz) has been shown to decrease the excitability of the motor cortex [16], whereas high frequency rTMS (≥ 5 Hz) produces the opposite effect [17]. rTMS causes activity changes in regions interconnected with the stimulated area through mono- or polysynaptic connections [18], modulates subcortical transmitter concentrations [19], and was shown to induce morphological modifications in stimulated areas as well as in areas linked to them [20]. Based on these findings, rTMS is used experimentally to treat a wide range of clinical disorders that may involve altered states of cortical excitability [21], such as major depression [22,23], auditory hallucinations [24], and stroke [25].

### TMS and tinnitus

Repetitive transcranial magnetic stimulation has recently been adopted to strengthen the concept of focally increased cortical excitability as a pathophysiological mechanism of tinnitus perception. Initially, it was shown that a high-frequency (10 Hz) rTMS induced 'virtual' lesion of temporoparietal cortical areas can transiently reduce tinnitus [26,27]. Further studies indicated that the effect size was negatively correlated with the tinnitus duration [28,29]. On the basis of these data, it has been proposed that a rTMS-induced reduction of hyperactivity of these cortical areas could yield beneficial effects for patients with chronic tinnitus [27]. To test this notion, low frequency rTMS was applied to areas of the temporoparietal cortex showing tinnitus-related hyperacitivity. Immediately after this intervention, tinnitus loudness was reduced for up to 30 min. The degree of reduction was dose dependent, and negatively correlated with tinnitus duration [11]. Several small clinical studies indicate that repeated application of low-frequency rTMS up to 2 weeks may have lasting ameliorating effects on chronic tinnitus [30-37]. However, the effect size was mostly moderate and interindividual responses as well as effect duration were highly variable. Further studies are needed to assess the clinical relevance of rTMS treatment in tinnitus and to identify the optimal stimulation paradigms as well as the most effective stimulation target site. Recently, continuous theta burst stimulation (cTBS) has been put forward as a modified rTMS paradigm [38] that reduces cortical excitability by applying three pulses at 50 Hz repeated every 200 ms over 40 s [38,39] at an intensity of 80% active motor threshold (AMT). For clinical purposes cTBS appears to be an applicable technique due to its low intensity, short duration, and similar efficacy [40]. In a first case report we demonstrated that cTBS is an effective approach to treat tinnitus [41].

### Objectives

We hypothesize that compared to sham stimulation cTBS can induce a distinctive attenuation of tinnitus distress and loudness and thus be of therapeutic value. Further aims of the study are:

• To compare the effect of cTBS on the secondary auditory cortex with its effect on the temporoparietal association cortex.

• Assessment of safety in terms of impairment in audiologic and speech-audiometric measures as well as the documentation of other unwanted side effects.

## Design and Methods

**Figure 1:**
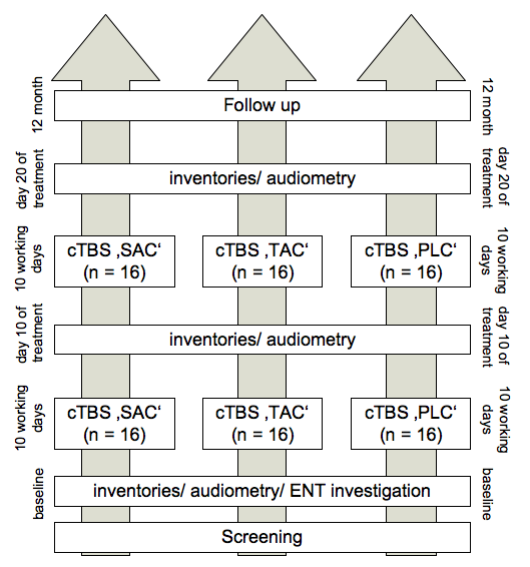
**Study Design**.

This randomised, placebo controlled study consists of 3 arms in a parallel design (Figure 1). Tinnitus distress, loudness and annoyance will be assessed before, during, and after 4 weeks of bilateral cTBS. Forty-eight patients will be randomised in an 1:1:1 ratio to either cTBS over the secondary auditory cortex (SAC), the temporoparietal association cortex (TAC) or sham stimulation (placebo: PLC).

### Population

The patient population will consist of patients with a wide middle range of tinnitus duration and excluding those with an increased risk for adverse effect of TBS.

#### Inclusion criteria

• Written informed consent

• Age: between 18 and 75 years

• Chronic tinnitus since at least 6 month, but not more than 5 years.

#### Exclusion criteria

• Objective tinnitus

• Acute or chronic inflammation of the middle ear, acute hearing loss, hearing loss due to an acoustic trauma within less than past 6 weeks

• Morbus Menière or fluctuating hearing loss

• Epilepsy

• Brain trauma

• Brain surgery

• Heart pacemaker

• Intake of anticonvulsants, antipsychotics, and regular intake of benzodiazepin

• Suicidality

• Other severe pathological chronic condition that might confound treatment effects or interpretation of data

• Pregnancy

• Participation in another clinical study regarding tinnitus within the past 3 months and during enrolment in our study.

#### Data sets

For evaluation of security all data gathered will be used and analyzed as treated. The analysis of the efficacy measures, especially with respect to the stimulus placement, requires a concentration on patients treated per protocol (PP). Missing observations are imputed using a full conditional Markov-Chain Monte Carlo method for variables with more than 75% valid values. An exploratory analysis of all dependent variables by the randomised treatment, i. e. the mere intention to treat (ITT), will be conducted to plan subsequent confirmatory studies.

### Screening

Patients will be recruited via announcement in the local press, the tinnitus outpatient clinic at the Department of Psychiatry and Psychotherapy, and the outpatient clinic of the Department of Otorhinolaryngology of the University Hospital of Tübingen. A psychiatrist and an otolaryngologist will assess the eligibility according to the inclusion and exclusion criteria listed above.

### Baseline assessments

The detailed time course and plan of the study is displayed in Table 1.

**Table 1:** Time course of the study

Investigation	Screening	Baseline	Treatment				Follow-Up				
			
			2 weeks		2 weeks		11 months				
Visit #	V0	V1	V2-10	V11	V12-20	V21	V22	V23	V24	V25	V26

informed consent	×										

demograph. data	×										

inclusion/exclusion criteria	×										

randomization		×									

ENT investigation/tinnitus localization		×									

audiogram/speech audiometry		×		×		×					

STI		×									

cTBS treatment SAC, TAC, PLC			×	×	×	×					

documentation of side effects			×	×	×	×					

TQ		×		×		x *	×	×	×	×	×

VAS (distress, loudness)		×	×	×	×	×	×	×	×	×	×

VAS (change)				×		×					

SCL-90R		×		×		×	×	×	×	×	×

BDI		×		×		×	×	×	×	×	×

*primary outcome measure

• Ear Nose and Throat (ENT) examination and audiological assessment including

- Standard pure tone audiometry

- Speech audiometry in quiet (mono- and multi-syllables, "Freiburger speech test")

- Hearing in noise test ("Oldenburger sentence test") [42]

- Audiological tinnitus matching

• Structered Tinnitus Interview (STI) [43]

• Tinnitus Questionnaire (TQ) [44,45]

• Visual Analogue Scales (VAS) for tinnitus loudness and annoyance [46]

• Beck Depression Inventory (BDI) [47]

• Symptom checklist 90-revised (SCL-90R) [48].

### Safety

Safety measures are pure tone and speech audiometry as well as speech understanding in noise tests (at Baseline, after 2 weeks of treatment and after the end of the treatment). Adverse events are assessed daily and documented according to the GCP guidelines. Safety and adverse events will be reported together with the results. The occurence of possible adverse effects will be reported in a case report form and handled in compliance with GCP. Study monitoring is provided by CenTrial (Tübingen, Germany).

### Outcome measures

The primary efficacy measure in the present study is the change of the tinnitus distress as measured by the TQ [45] after 4 weeks of cTBS. The TQ [45]), originally developed by Hallam et al. [49], is the most widely used, well validated and reliable inventory [50] to quantify tinnitus ditress available in German language [30,32,34,41,51-58]. It comprises 52 multi-scaled items in order to assess emotional and cognitive handicaps, penetrance of the tinnitus, hearing problems, sleep disturbances, and somatic discomfort. The TQ was used in pharmacological, behavioural and TMS studies. Pharmacological studies did not yield therapeutic effects [58,59]. Behavioural therapy yield effect sizes between 6 and 28% on the TQ [60,61]. Pilot rTMS studies reported effects sizes between 5 and 25% [34,41,52].

Secondary efficacy variables are tinnitus change [46] and the changes in BDI [47] and in the VAS for tinnitus loudness and tinnitus annoyance [46,62].

Changes regarding psychopathology will be measured using the SCL-90R [48]. Feasibility of the study will be assessed and reported together with the results.

### Follow-up assessments

Duration of treatment effects will be measured (TQ, VAS, BDI) 6, 8, 16, 28, and 56 weeks after the first stimulation. Furthermore, 6 months after the end of treatment patients will be asked to report if they regard the estimated benefit as worth the effort.

### Withdrawal from the study

In case of endangerment of personal security or lack of compliance or withdrawal of informed consent, a patient will instantly be excluded from further participation in the study.

### Sample size calculation

Effect size and dispersion were taken from our last study of TMS against tinnitus [34]. The ratios of tinnitus distress score after treatment to tinnitus distress score at baseline are assumed to follow normal distributions with standard deviation 0.18 after taking logarithms. A relevant effect size of 0.19 logarithmic units would arise from the ratio of geometric mean scores of 80% of baseline after efficacious stimulation to 97% after sham stimulation. All three pairwise group comparisons will be conducted. Therefore the probabilities of the errors of first and second kind are Bonferroni-adjusted to significance level 0.017 and power 0.93 locally to ensure multiple level 0.05 and multiple power 0.8. Consequently the planned number of patients to be included in the study is 48, i. e. 16 in each arm. A closed testing procedure preserves the multiple significance level and power while treatments are compared at different points in time.

### Data analysis

The geometric mean tinnitus score (TQ) in percent baseline will be compared between the three groups after the full treatment course of 4 weeks after the first stimulation, after 6 weeks, as well as 28 and 56 weeks after the first cTBS treatment in that prespecified order, as we expect effects sizes to have that order. If one group comparison is not statistically significant, the following comparisons are rated as not significant too. The confidence intervals for ratios of geometric means of tinnitus scores at 4 and 6 weeks will be computed from an analysis of variance (ANOVA) with factors *group *(SAC, TAC, PLC) and *time *(4 and 6 weeks) and the group*time interaction. Further ANOVAs of TQ with the between subject factor *group *will be carried out after 8, 28, and 56 weeks after the first treatment. Their residual variances are expected to differ. The longitudinal course of the stimulation effect will be described using all TQ measurements, including measurements at 2 and 16 weeks. Secondary efficacy measures will be analysed similarly. Only the tinnitus change cannot be divided by a baseline value and will be analysed by ordinal logistic regression as it is ordinal. The daily measurements on visual analog scales will be shown in diagrams. Confidence interval computations will assume normality of logit-transformed VAS. The routinely sampled safety data will all be plotted for each patient and will be used to quantify intensity and duration of adverse effects should such occur. Data analysis will be performed in the Department of Medical Biometry of University Hospital of Tübingen using JMP 7.0 (SAS Institute Inc.) after imputation with SPSS 17.0 (SPSS Inc.).

### Randomization

A randomisation list was prepared by the Department of Medical Biometry of University Hospital of Tübingen (RV) using permuted blocks. Allocations were concealed in opaque sealed envelopes, that are opened by a third person immediately prior to the first treatment.

### Treatment

After screening, written informed consent, physical ENT examination and audiological assessments, patients will be assigned to one of the real (SAC, TAC) or sham (PLC) treatment groups. TMS will be applied using a Magstim Super Rapid (The Magstim Company Ltd, Whitland, UK) with a figure-eight coil (diameter of each winding: 70 mm, biphasic stimulus of 250 μs, peak magnetic field: 2T). The individual motor threshold (AMT) will be assessed at the beginning of the first treatment session. AMT is defined as minimum stimulation intensity required to induce a motor evoked potential (MEP) of more than 200 μV on at least 5 out of 10 trials from the contralateral abductor pollicis brevis muscle (APB) while maintaining a voluntary contraction of about 20% of maximum [39]. Stimulation (cTBS) intensity is 80% AMT and will be applied to each side for 40 s in alternating order. Fifteen minutes after the first two trains, a second pair of cTBS will be applied (a total of 2400 stimuli per day). Patients receive cTBS treatment each working day for 4 weeks.

### Blinding

The endpoint assessor remains masked to the treatment until the final data analysis. For adequate masking of the patients, sham stimulations will be performed at the lower occiput in the same distance to the ear [11,27,63]. In this way, the control stimulation is accompanied by a similar noise (between 60 and 75 dB) and comparable aversive sensation (pricking, muscle twitches).

### Data Management

Data are collected on paper case report forms that are stored in a safe place until 10 years after completion of the trial. Data are entered directly in the statistical analysis software file on a safely kept computer with individual user passwords. This process is replicated by a second person and the resulting files are compared regularly, so that ambiguous entries can be questioned in short time. Plausibility checks, source data verification and quality control are carried out by an external monitor.

### Ethics, Consent, Study Organization and Registration

The trial will be conducted in agreement with the principles of the Declaration of Helsinki, and with the guidlines of Good Clinical Practice (GCP) of the International Conference on Harmonisation of Technical Requirements for Registration of Pharmaceuticals for Human Use (ICH). The protocol was approved of by the local Indpendent Ethics Committee (Institutional Review Board). Funding is provided by the German Research Council. The investigator will explain the benefits and risks of participation in the study to each subject and will provide an informed consent form approved by the independent ethics committee. Only patients, who sign the form, will be included in the study. Results will be published so that patients cannot be identified. All eligible patients are seen by a psychiatrist and an otolaryngologist and are enrolled after giving informed consent. All findings will be recorded in the patients' medical records and the CRF provided for this study. Study auditing, CRF compilation and study monitoring is performed by CenTrial GmbH (Tübingen, Germany).

## Discussion

The available data on the efficacy of tinnitus treatment with rTMS is incomplete [30-37]. The effect size, the clinical relevance and the optimal stimulation parameters are virtually unknown. This placebo-controlled phase II clinical trial has been designed to investigate the efficacy of a 4 weeks treatment with bilateral cTBS on chronic tinnitus and to compare the effectiveness of two different stimulation areas. Since the laterality of tinnitus-related cortical hyperactivity and rTMS effects in mono- and bilateral tinnitus have been shown to be interindividually variable [11,64], we opted for a bilateral cTBS. Recent studies on therapeutic efficacy of rTMS in major depression pointing towards a superior effectiveness of longer treatment durations [23] prompted us to extend cTBS treatment to 4 weeks. Moreover, against the background of a dose dependency of rTMS effects in tinnitus [11] and in order to assure a sufficient dose of stimulation, the patients will receive two stimulation trains on each side. Hence, bilateral, long-term application of cTBS to two different cortical sites will provide comprehensive data for an evaluation of the clinical relevance of TMS treatment in chronic tinnitus and an improvement of treatment parameters. Based on the effect size and standard deviation of the Intention-to-Treat analysis as well as on the finding of the optimal stimulation localization a larger clinical efficacy study will be designed.

## Abbreviations

APB: abductor pollicis brevis muscle; AMT: active motor threshold; BDI: Beck Depression Inventory; ITT: intention to treat; LOCF: last observation carried forward; MEP: motor evoked potential; MT: motor treshold; PLC: placebo (sham stimulation); SAC: secondary auditory cortex; SCL-90R: Symptom Checklist 90-revised; TAC: tertiary auditory cortex; TQ: Tinnitus Questionnaire; VAS: Visual Analogue Scale

## Competing interests

The authors declare that they have no competing interests.

## Authors' contributions

All authors contributed to the study design. CP is the initiator and principal investigator of the study. RV is the trial statistician, and carried out the randomisation and sample size calculations. CA is the trial manager. CP, CA, and SP drafted the original study protocol. The manuscript was written by CP, CA, and RV. All authors have read and approved the final manuscript.
